# Characterizing Myasthenia Gravis Symptoms, Exacerbations, and Crises From Neurologist's Clinical Notes Using Natural Language Processing

**DOI:** 10.7759/cureus.65792

**Published:** 2024-07-30

**Authors:** Jonathan D Darer, Jacqueline Pesa, Zia Choudhry, Alberto E Batista, Purva Parab, Xiaoyun Yang, Raghav Govindarajan

**Affiliations:** 1 Medical and Innovation, Health Analytics, Clarksville, USA; 2 Real World Value and Evidence, Immunology, Janssen Scientific Affairs, Titusville, USA; 3 Rare Antibody Diseases, Janssen Scientific Affairs, Titusville, USA; 4 Global Market Access, Janssen Scientific Affairs, Titusville, USA; 5 Biostatistics, Health Analytics, Clarksville, USA; 6 Neurology, Cleveland Clinic Flo, Weston, USA

**Keywords:** myasthenia gravis exacerbations, clinical progress notes, natural language processing, myasthenia gravis crisis, myasthenia gravis

## Abstract

Background

Myasthenia gravis (MG) is a rare, autoantibody neuromuscular disorder characterized by fatigable weakness. Real-world evidence based on administrative and structured datasets regarding MG may miss important details related to the clinical encounter. Examination of free-text clinical progress notes has the potential to illuminate aspects of MG care.

Objective

The primary objective was to examine and characterize neurologist progress notes in the care of individuals with MG regarding the prevalence of documentation of clinical subtypes, antibody status, symptomatology, and MG deteriorations, including exacerbations and crises. The secondary objectives were to categorize MG deteriorations into practical, objective states as well as examine potential sources of clinical inertia in MG care.

Methods

We performed a retrospective, cross-sectional analysis of de-identified neurologist clinical notes from 2017 to 2022. A qualitative analysis of physician descriptions of MG deteriorations and a discussion of risks in MG care (risk for adverse effects, risk for clinical decompensation, etc.) was performed.

Results

Of the 3,085 individuals with MG, clinical subtypes and antibody status identified included gMG (n = 400; 13.0%), ocular MG (n = 253; 8.2%), MG unspecified (2,432; 78.8%), seropositivity for acetylcholine receptor antibody (n = 441; 14.3%), and MuSK antibody (n = 29; 0.9%). The most common gMG manifestations were dysphagia (n = 712; 23.0%), dyspnea (n = 626; 20.3%), and dysarthria (n = 514; 16.7%). In MG crisis patients, documentation of difficulties with MG standard therapies was common (n = 62; 45.2%). The qualitative analysis of MG deterioration types includes symptom fluctuation, symptom worsening with treatment intensification, MG deterioration with rescue therapy, and MG crisis. Qualitative analysis of MG-related risks included the toxicity of new therapies and concern for worsening MG because of changing therapies.

Conclusions

This study of neurologist progress notes demonstrates the potential for real-world evidence generation in the care of individuals with MG. MG patients suffer fluctuating symptomatology and a spectrum of clinical deteriorations. Adverse effects of MG therapies are common, highlighting the need for effective, less toxic treatments.

## Introduction

Myasthenia gravis (MG) is a rare, chronic autoimmune neuromuscular autoantibody-driven disorder characterized by fatigable weakness and unpredictable exacerbations [1-4]. MG has an overall prevalence of 150-250 cases per million worldwide [5,6]. Common symptoms include ptosis, diplopia, dysphagia, difficulty chewing, dysarthria, extremity weakness, axial weakness, general fatigue, and generalized weakness [2,7-10]. Symptom severity varies widely, ranging from daily mild fluctuating weakness to severe respiratory muscle weakness and ventilatory failure [11-13]. Historically, myasthenia gravis has been more common in women, who tend to present at age 20-40 [14,15], while men tend to present later in life between 60 and 80. Up to 20% of individuals suffer from myasthenic crises over the course of their illness, requiring mechanical ventilation due to an inability to maintain their airway and subsequent respiratory failure [3,10,16,17].

There is no cure for MG, and therapeutic approaches for MG are individualized based on clinical subtyping, including age of onset, severity of disease, patient serotype, and comorbidities aimed at managing symptoms and preventing clinical events [6,10,18-22]. A substantial number of individuals with MG experience variable or slow onset of treatment benefits, medication side effects, failure to respond to one or more medications, or require long-term immunosuppression [7,23,24]. Approximately 15-20% of patients suffer from refractory MG and are not responding to available treatments [6,7,10,12,13,23,25-29]. The experience of living with MG can be challenging, often impacting mental health, social functioning, and work activities [30,31].

The physician-patient encounter remains the primary setting for MG care, yet little is known about what occurs during these encounters. The application of natural language processing (NLP) methods to clinical notes is an active area of research and has been applied to identify symptoms, conditions, and laboratory results, as well as generate digital patient phenotypes [32-35]. NLP has been used to study neurologic disorders, including stroke, transient ischemic attack, epilepsy, headaches, multiple sclerosis, and cerebral aneurysms [36,37]. To our knowledge, NLP has not been previously used to study myasthenia gravis.

To better understand care delivery during the clinical encounter and the language used by physicians to describe MG-related phenomena, we performed a mixed-methods study of neurologist clinical notes using NLP and qualitative analysis of physician documentation.

To better understand care delivery during the clinical encounter and the language used by physicians to describe MG-related phenomena, we performed a mixed-methods study of neurologist clinical notes using NLP and qualitative analysis of physician documentation.

Study objectives

The objectives of this study were to analyze neurologist clinical notes and examine physician language, clinical events, and challenges in managing myasthenia gravis. Specifically, the primary objective was to use NLP to assess provider documentation of details of the patient's MG condition (i.e., clinical subtypes, antibody status), symptoms (e.g., diplopia, dysphagia), and disease states (worsening, exacerbation, crisis). Additionally, this study sought to examine the experiences of individuals with a history of MG crises, specifically their negative experiences with MG therapies. Lastly, this study sought to examine neurologist documentation of MG acute exacerbations to identify meaningful classifications that could be used to track clinical outcomes.

## Materials and methods

We performed a retrospective, cross-sectional analysis of de-identified neurologist clinical notes from 2017 to 2022. The analysis was conducted on a medical transcription dataset, Amplity Insights, which includes full-text transcripts of dictated clinical notes covering 150,000 physicians from private practice, integrated delivery networks, and academic institutions from over 40,000 clinics and hospitals, with representation across all 50 U.S. states. The dataset includes initial office consultations, follow-up visits, urgent care visits, emergency department and hospital admissions and discharges, postoperative consultations, office notes, and referral letters. This study was limited to the analysis of de-identified clinician notes and did not involve the collection of data from or engagement with human subjects.

Identification and selection of eligible notes

The data licensed included 45,414 records that included the term “myasthenia gravis.” Of these 45,414 notes, 6,557 were written by a neurologist. Eligibility criteria for the analyzable notes included only one patient per text file (37 multi-patient text files were excluded), NLP analysis-ready structure and format (416 notes with unusual formats, including carriage returns in the middle of sentences or notes without any structure, were excluded), and a positive assertion of MG (923 notes with laboratory orders related to MG, suspected diagnoses, family history of MG, and referential discussions of MG were excluded). The final analysis dataset included 5,183 notes attributable to 3,085 patients.

NLP model development

A rules-based NLP model was developed using spaCy, an open-source library for NLP, to create an analyzable dataset focused on MG-related clinical phenomena, including clinical subtypes, disease status, and symptoms. The NLP model development consisted of four major steps, including a) knowledge framework, b) tokenization and named entity recognition, c) assertion status and clinical status detection, and d) section-specific analysis (Supplemental Material A).

Knowledge framework

A term and phrase vocabulary of MG-related symptoms, medications, procedures, laboratory testing, clinical events, and physiology terms was developed based on an iterative review of notes and discussion with the authors. Relevant MeSH headings were also reviewed. Example terms and phrases included respiratory symptoms (e.g., dyspnea, respiratory distress, and respiratory failure), bulbar symptoms (e.g., dysphagia, dysarthria, tongue weakness, and oropharyngeal weakness), appendicular symptoms (e.g., lower extremity weakness, upper extremity weakness), and ocular symptoms (e.g., ptosis, diplopia) Standard MG treatments included pyridostigmine, azathioprine, corticosteroids, IVIg, plasmapheresis, rituximab, eculizumab, tacrolimus, mycophenolate, methotrexate, and cyclosporine (Table 6 in the Appendices).

Tokenization and named entity recognition

To facilitate NLP rule development for MG symptoms, a mapping of synonymous terms and phrases was developed. For example, “eyelid droop” and “drooping eyelids” were mapped to "ptosis." The listing of synonyms was iterated over a review of hundreds of notes during development (Table 7 in the Appendices). SpaCy was used to consume sentences and annotate each word or phrase as a named entity or cue to prepare for the application of the MG NLP model. 

Assertion status and clinical status detection

To clarify the meaning of a symptom or condition within the context of a sentence, assertion status detection rules were developed. Types of assertion status included “negation” (e.g., “no dysphagia"), “hypothetical” (e.g., “possible MG crisis”), and “family history” (e.g., “father with a history of MG”). Similarly, to clarify the trajectory of symptoms within the context of a sentence, clinical status rules were developed. Types of clinical status included “improving” (e.g., “ptosis is getting better”), and “worsening” (e.g., “dysphagia is increasing”). Cue terms and phrases for each of the assertion status and clinical status categories were identified (Table 8 in the Appendices). 

MG NLP rules were developed iteratively using the named entity lists, cue term lists, and logical expressions to perform assertion status and clinical status detection. These rules were then applied to sentences to generate a final analytical dataset.

NLP assessment

**Figure 1:**
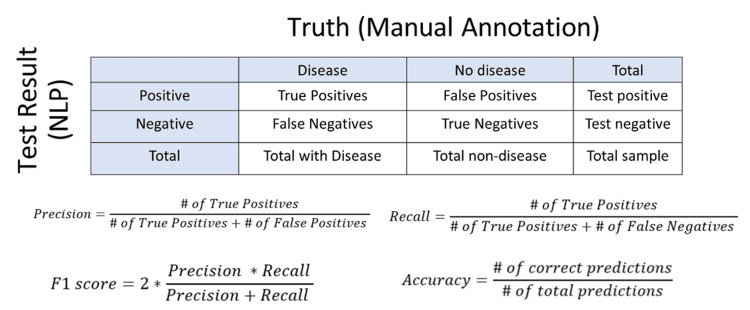
Definitions of accuracy, recall, precision, and F1 score using a confusion matrix

The NLP model's performance with regards to correctly detecting assertion status (negation, hypothetical, family history, and positive assertion) was assessed for accuracy, recall, precision, and the F1 score (a balanced measure of the model’s performance regarding recall and precision) (Figure 1 in the Appendices). Because the dataset contained large amounts of content not relevant to this study, model performance was assessed against 16 datasets of 50 randomly selected sentences containing at least one mention of a concept of interest, including dysphagia, ptosis, generalized myasthenia gravis, myasthenia gravis exacerbation, or crisis. Each of the 16 datasets was then manually annotated using Prodigy, a proprietary software application, to create a gold-standard dataset, and the results were compared with the NLP model applied to the same sentences (Supplemental Material B). 

MG crisis identification and abstraction

MG crisis was identified with a two-step process beginning with a search to identify notes using the NLP results that contained positive assertions for MG crisis or notes containing a positive assertion of “respiratory failure," “mechanical ventilation," "intubation," “bilevel positive airway pressure," or "BIPAP." All candidate charts were manually reviewed to confirm the history of a current or past crisis or the use of mechanical ventilation caused by MG (i.e., not related to COPD or other respiratory conditions). Patients with a confirmed history of MG crises had all notes reviewed and abstracted for further analysis by an internal medicine physician (JD). A framework was developed and used in the abstraction that focused on triggering events, and problems with MG medications, including adverse effects, interactions with comorbidities, and difficulties with access or availability.

Qualitative analysis of physician language of acute MG decompensation and risks

To examine the language used by physicians in the care of MG, one of the physician authors (JD) identified candidate concepts for clinical events, including MG-related symptoms, deteriorations, and crises, which were then reviewed by the authorship team, including an expert in neuromuscular medicine. Terms and phrases were normalized to major concepts to assist with NLP concept identification (Table 7 in the Appendices).

The thematic grouping of physician documentation regarding clinical deteriorations and MG-related risks was performed over three one-hour sessions over a six-week period. Existing frameworks for clinical severity were reviewed in advance of the sessions, including the Myasthenia Gravis Foundation of America and International Guidelines [22,38].

Analysis

Demographic statistics (age, sex) were calculated based on the Amplity dataset meta-data. Percentages of symptoms, clinical subtypes, and clinical events found in clinical notes were calculated at the patient level. The analysis of physician language was grouped by theme and visualized with representative quotes.

## Results

NLP performance

The NLP model’s performance to detect assertion status (negation, hypothetical, family history, and positive assertion) was characterized by an accuracy of 0.96, precision of 0.98, recall of 0.98, and F1 score of 0.97 (Table 9 in the Appendices).

Demographics and clinical subtypes

The reported age of individuals with MG was 65 or greater (n = 1,083; 35.1%), 40-64 (n = 599; 19.4%), less than 40 (n = 172; 5.6%), and missing (n = 1,231; 39.9%). The sex of the patients was female (n = 1,550; 50.2%), male (n = 1,526; 49.5%), and missing (n = 9; 0.3%). Neurologist documentation did not reference clinical subtypes in 78.8% of notes. Of the remaining notes, the most frequently documented clinical subtype was generalized (n = 400; 13.0%) and ocular (n = 253; 8.2%). MG antibody status was not documented in 75.5% of records, with the most common documented status being seropositivity for acetylcholine receptor antibodies (AChR, n = 441; 14.3%), followed by seronegativity (n = 224; 7.9%), seronegativity for muscle-specific tyrosine kinase (MUSK, n = 45; 1.4%), and seropositivity for anti-MuSK antibodies (n = 29; 0.9%) (Table 1). 

**Table 1:** Note attrition **Amplity notes that were formatted with frequent line breaks in the middle of sentences or no line breaks at all without spacing or structure were excluded from the analysis. MG: Myasthenia gravis, NLP: Natural language processing

Description of notes	Notes removed	Number of notes remaining
All available notes that were labeled “myasthenia gravis” or “MG” by Amplity Insights	0	45414
Neurology notes	-	6557
Notes limited to a single patient and encounter	37	6520
Notes limited to formatting compatible with NLP analysis**	416	6104
Notes limited to patients with confirmed diagnosis of MG	921	5183

Clinical events

Documentation of MG clinical events included exacerbations (n = 391; 12.7%), crises (n = 134; 4.3%), and worsening or fluctuating symptoms (n = 138; 4.5%) (Table 2). The most common symptom was ptosis (n = 1,071; 34.7%), followed by diplopia (n = 926; 30.0%), dysphagia (n = 712; 23.1%), fatigue (n = 693; 22.5%), dyspnea (n = 626; 20.=3%), dysarthria (n = 514; 16.67%), generalized weakness (n = 350; 11.3%), respiratory distress or failure (n = 293; 9.5%), extremity weakness (n = 280; 9.1%), face-bulbar weakness (n = 232; 7.5%), and neck weakness (n = 121; 3.9%) (Table 2). The use of formal instruments to assess MG symptoms, such as the Myasthenia Gravis-Activities of Daily Living (MG-ADL), was found in less than 2% of notes. 

**Table 2:** MG patient attributes were found using NLP MG: Myasthenia gravis, MuSK: Muscle-specific tyrosine kinase, NLP: Natural language processing

Population Attribute	MG patients (n = 3,085)
	n (%)
Age	
Less than 40	172 (5.6%)
40-64	599 (19.4%)
65 or greater	1,083 (35.1%)
Missing	1,231 (39.9%)
Sex	
Female	1,550 (50.2%)
Male	1,526 (49.5%)
Missing	9 (0.3%)
Clinical Subpopulation	
Unspecified	2,432 (78.8)
Generalized MG	400 (13.0)
Ocular	253 (8.2)
MG Serology status	
Seropositive for acetylcholine receptor antibody	441 (14.3)
Seronegative	224 (7.9)
MuSK antibody negative	45 (1.4)
Seropositive for MuSK antibody	29 (0.9)
MG disease status	
Exacerbations	391 (12.7)
Worsening	138 (4.5)
Crises	134 (4.3)
MG symptoms	
Ptosis	1,071 (34.7)
Diplopia	926 (30.0)
Dysphagia	712 (23.0)
Fatigue	693 (22.5)
Dyspnea	626 (20.3)
Dysarthria	514 (16.7)
Generalized weakness	350 (11.3)
Respiratory distress or respiratory failure	293 (9.5)
Extremity weakness	280 (9.1)
Face, bulbar, tongue, or oropharyngeal weakness	232 (7.5)
Neck weakness	121 (3.9)
Any symptom reported as worsening	1170 (37.9)

MG crisis

Of the 137 patients who were identified with MG crises, 100 notes (73.0%) explicitly used the term "crisis,” with an additional 37 (27.0%) patients identified by mechanical ventilation (either via intubation or non-invasive) specifically due to MG-related weakness. A subset of 19 patients (13.9%) were documented with two or more crises, of which 11 (8.0%) had recurrent crises separated by fewer than four weeks. Reports of crisis triggers were present for 32 (23.3%) of patients. The most documented trigger was pneumonia (n = 13; 9.5%), followed by UTI (n = 5; 3.6%). Other infectious causes included sepsis, osteomyelitis, diverticulitis, or a non-specific mention of infection. Reports of difficulty with standard MG therapies (Table 10 in the Appendices) were reported for 62 (45.2%) of patients with MG crises, including 44 (32.1%) with adverse effects, 16 (11.7%) with limited response, 11 (8.0%) with interaction or potential interaction with comorbidities, 3 (2.2%) with difficulty getting access to a therapy due to insurance or prior authorization, and three (2.2%) related to poor availability at treatment facilities. The treatment most associated with neurologist-documented treatment difficulty was pyridostigmine (n = 27; 19.7%), followed by IVIG (n = 23; 16.7%), corticosteroids (n = 19; 13.8%), plasmapheresis (n = 12; 8.8%), azathioprine (n = 12; 8.8%), mycophenolate (n = 1; 0.7%), eculizumab (n = 1; 0.7%), and rituximab (n = 1; 0.7%) (Table 3).

**Table 3:** Characteristics of the 137 patients identified with an MG crisis *Standard MG treatments include pyridostigmine, azathioprine, corticosteroids, IVIg, plasmapheresis, rituximab, eculizumab, tacrolimus, methotrexate, cyclosporine ‡Neurologists may have documented challenges with multiple agents for a single patient and multiple challenges with a single agent. Please see Table 10 in the appendices for further details. *MG: Myasthenia gravis, NLP: Natural language processing, UTI: Urinary tract infection, URI: Upper respiratory infection, IVIg: Intravenous Immunoglobulin Therapy

MG crisis documentation attributes	Patients (n = 137)
	n (%)
Documentation of MG with term “crisis”	100 (73.0)
Documentation of MG and mechanical ventilation without “crisis” term	37 (27.0)
Recurrent crises	19 (13.9)
Recurrent crises separated by less than 4 weeks	11 (8.0)
MG crisis trigger (any)	32 (23.3)
Pneumonia	13 (9.5)
UTI	5 (3.6)
Other infection (sepsis, osteomyelitis, diverticulitis, URI)	11 (8.0)
Surgery	1 (0.7)
Hip fracture	1 (0.7)
Influenza vaccination	1 (0.7)
Challenges or difficulty receiving MG standard therapies*	62 (45.2)
Adverse effects	44 (32.1)
Limited response	16 (11.7)
Interaction or potential interaction with co-morbidity	11 (8.0)
Problems with insurance or prior authorization	3 (2.2)
Treatment with poor availability at treatment facility	3 (2.2)
Challenges or difficulty receiving specific MG therapy‡	-
Pyridostigmine	27 (19.7)
IVIg	23 (16.7)
Corticosteroids	19 (13.8)
Plasmapheresis	12 (8.8)
Azathioprine	12 (8.8)
Mycophenolate	1 (0.7)
Eculizumab	1 (0.7)
Rituximab	1 (0.7)

Thematic analysis of clinical events

Thematic analysis of physician sentences used to describe MG clinical events yielded the following categories: symptom fluctuations occurring within 24 hours identified by phrases such as *“worse in the afternoons and evenings”* or *“gets worse as the days go by.”* MG deteriorations were categorized into three categories: *"Symptom deterioration with the intensification of therapy,"* *"Deterioration with hospitalization or rescue therapy,"* and *"MG crisis or deterioration requiring mechanical ventilation."* Physician discussions of their assessments of MG patients that included uncertainty were characterized as “suspected MG deteriorations or crisis” (Table 4).

**Table 4:** Physician text examples describe MG clinical events, including symptoms, exacerbations, and crises *MG: Myasthenia gravis, IVIg: Intravenous immunoglobulin therapy, FVC: Forced vital capacity

MG Symptoms and Events	Description	Representative Text Examples
MG symptom fluctuations	Changes to specific symptoms within 24 hours that do not require intensification of treatment.	"He states the dysphagia is worse in the afternoons and evenings."
		"She states that ptosis is not so severe in the morning; however, it gets worse as the day goes by."
		"This ptosis also varies in intensity; sometimes it is more, sometimes it is less prominent and it can happen at any time of the day or night. “
MG symptom worsening with treatment intensification	Changes in MG status or symptoms that require additions or modifications to treatment but do not require admission to the hospital or rescue therapy (i.e., IVIg or plasmapheresis).	“With increasing problems with dysphagia and dysarthria, she was started on prednisone 10mg daily.”
	Symptom worsening terms include: “worsening”, “increasing”, “progressing”, “aggravating”	“He has episodic increases in diplopia and is utilizing a predominantly prednisone to treat his disease.” “Based on the development of unequivocal extremity weakness, I started her on prednisone and she was recently up to 30 mg q.d.”
MG deterioration with rescue therapy (i.e., IVIg or plasmapheresis) or hospitalization	Changes in MG status or symptoms that require hospitalization or rescue therapy (i.e., IVIg or plasmapheresis).	"This is an 82-year-old gentleman with diabetes, hypertension, hyperlipidemia and anemia, also has myasthenia gravis, which was treated last month with plasmapheresis for an exacerbation in which the patient developed difficulty swallowing and visual disturbance."
	MG status deterioration phrases include: “exacerbation”, “flare”, “out of control”, “deterioration”, “decompensation”, “uncontrolled”.	“Myasthenia gravis exacerbation: no need for intubation at this time, fvc 1.1 liters, able to communicate in full sentences, we will discontinue prednisone and continue IVIg, currently day 2 of 5.”
		"She did receive IVIg this past year for a myasthenic flare."
		"A 29-year-old female admitted with exacerbation of bulbar myasthenia with respiratory insufficiency." "Looking through her medical record, she has been hospitalized nearly every month secondary to exacerbations of her myasthenia gravis."
MG crisis or deterioration requiring mechanical ventilation	Deterioration of MG requiring mechanical ventilation. MG status deterioration phrases include “crisis”	“Impression: a 50-year-old gentleman with refractory myasthenia gravis resulting in recurrent episodes of respiratory failure requiring intubation and mechanical ventilation.”
		"Reason for consultation: worsening myasthenia gravis requiring mechanical ventilation."
		“The patient has a history of refractory myasthenia gravis with several recent myasthenia gravis crises.”
		"Myasthenia gravis, in crisis with acute respiratory failure status-post intubation and extubation."
Suspected MG deteriorations and crises	Concern for deterioration of MG status due to presenting symptoms but requiring more time or testing to be certain.	"Generalized weakness, suspected myasthenia gravis exacerbation with the potential for crisis."
	Suspected MG deterioration cues include a combination of MG deterioration terms and suspected cues including: “probable”, “possible”, “suspected”, “differential diagnosis”, “suggestive of”, “monitor for”, “impending”	"Shortness of breath is concerning for possible exacerbation of myasthenia gravis."
		"However, an exacerbation of myasthenia gravis could not be ruled out completely."
		“Impression: Myasthenia gravis, possibly going into crisis, because her treatment was delayed for almost 2 to 3 weeks.”
		"The impression is that of this gentleman with myasthenia gravis concern for impending myasthenia crisis.”
		"At this point, he is also being worked up for the possibility of pulmonary effusion as well as possibility of pneumonia which could be contributing factors although i suspect that he is having a myasthenic crisis underlying at all."

Physician language, MG-related risks, and potential for clinical inertia

Neurologist discussions of MG-related risks were categorized into four groups: *“Risk for changing therapy due to potential for toxicity and adverse side effects of new agents,”* *“Risks related to toxicities of existing MG treatments,”* *“Risk of changing existing MG treatments due to potential for worsening MG,”* and *“Risk for not managing MG”* (Table 5). Neurologist discussion or risks related to the toxicities of new or existing MG standard therapies ranged from short-term adverse effects, including increased respiratory secretions (5.8%), heart rhythm abnormalities (4.4%), decreased blood counts (4.4%), and renal failure (3.6%), as well as long-term effects related to corticosteroids and immunosuppression, including hyperglycemia (2.9%) and osteoporosis (1.5%). Discussions of reducing corticosteroids or other immunosuppression therapy were sometimes accompanied by neurologist and patient concerns regarding the potential for MG relapse and crisis, sometimes related to previous experience with deteriorations after corticosteroid taper. Neurologist concerns regarding adherence to therapy or non-treatment were often articulated in terms of the risk of MG deterioration, including life-threatening complications (Table 5).

**Table 5:** Physician language, risks of therapy, and potential sources of clinical inertia *MG: Myasthenia gravis, IVIg: Intravenous immunoglobulin therapy

Discussions of MG-Related Risks	Representative Text Examples
Risk for changing therapy due to potential for toxicity and adverse side effects of new agents	“Again, from the point of view of his myasthenia, he is doing quite well. I do not think the risk of immunosuppressive agents, corticosteroids or even IVIG, there is a nationwide shortage of this material, are warranted by his relatively minor myasthenia complaints. Most of them are the more vague fatigue and just not as strong as he used to be and I think that that would not be a good indication for putting more toxic agent on. My advice is that he stay with the Mestinon.” “I do not think he will be a good candidate for the Soliris because of the difficulties with the pancytopenia and the risk of concomitant infection with the complement inhibitor superimposed upon his chemotherapy.” IVIg would not be recommended, especially due to high risk of hypercoagulation on IVIg in a patient who is actively suffering from pulmonary embolism, recently diagnosed about 2-3 weeks ago and on coumadin.
Risks related to toxicities of existing MG treatments	“This may increase his risk for recurrent malignancy if we are to continue azathioprine.”
	“The patient is well aware about the risks of prednisone including avascular necrosis of the hips and our goal was to have the prednisone discontinued as soon as it is possible.” “We may need to consider an alternate immunosuppressive agent such as azathioprine or mycophenolate for maintenance therapy to minimize longer term glucocorticoid toxicity, for which he remains at risk for.”
Risk of changing existing MG treatments due to potential for worsening MG	“Reportedly, seropositive myasthenia gravis, on solid immunomodulatory therapy requiring refills. Has not had exacerbation in the last year. I will recommend to continue the course with a combined Imuran and prednisone. I would like to know what the Hematology has to say with regards to the Imuran. Also, any changes will certainly put her at risk of crisis.”
	“We discussed decreasing the prednisone and other prednisone sparing activities, but he said he really is very comfortable now compared to especially when he first got it and he does not want to change things despite the possible risk of cataract opportunistic infections, aseptic necrosis of the hip, and osteoporotic fractures.” “Certainly should he feel the imuran to be a risk, we could consult about possibly using another agent, though the patient has had myasthenia relapses when not being treated with imuran in the past.”
Risk for not managing MG	I apprised him of the risk of leaving his generalized myasthenia untreated including respiratory arrest and myasthenic crisis, which could be fatal. The patient was strongly urged to stay compliant with her prednisone and mestinon for myasthenia_gravis and is fully aware of risks of not doing so.

## Discussion

To better understand the care experience of individuals with MG and the challenges faced by practicing neurologists, we applied advanced analytic techniques as well as qualitative methods to clinical progress notes, which contain details not available in structured datasets. This investigation, the first of its kind, sought to assess the feasibility of extracting MG clinical subtypes and relevant symptoms from physician documentation and, at the same time, characterize the language used to describe MG clinical events as well as explore some of the challenges with medical decision-making and treatment in the care of individuals with MG.

Based upon the progress notes analyzed in the study, most neurologists describe the extent of myasthenia gravis through a description of clinical symptoms rather than using the terms “generalized” or "ocular." Neurologist notes frequently describe the presence, absence, characteristics, and trajectory of MG symptoms (e.g., ptosis, diplopia, dysphagia, and dysarthria). While numerous MG assessment instruments have been developed and validated for clinical research, including the myasthenia gravis activities of daily living (MG-ADL), myasthenia gravis disability scale, and myasthenia gravis quality of life 15 (MG-QoL15r), in our analysis, the use of these instruments was uncommon.

In this study, the language used by neurologists to describe clinical deterioration events ranged from mild fluctuations or worsening disease to more serious and substantial exacerbations of disease. to describe significant MG deterioration events including "exacerbation," "flare," "relapse," "deterioration," "decompensation," and “out of control” (Table 4). In contrast to this real-world clinical documentation, within the published literature, researchers have defined exacerbations using several different and more exacting methods. In a recent study of resource utilization, Phillips et al. used the ICD codes G70.01 (ICD-10) or 358.01 (ICD-9) to define MG exacerbations. The MGFA defines MG exacerbations and flares as worsening muscles throughout the body, but assistance is not required for breathing (i.e., presumably excluding MG crises); these may include worsened double vision, slurred speech, increased arm weakness, falling, unsteady walking, and difficulty swallowing [38]. Other studies have used multiple criteria, including hospitalization, the use of rescue therapy such as IVIg or plasmapheresis, and changes in the quantitative MG score [3,39].

More serious deteriorations associated with respiratory weakness and mechanical ventilation are commonly and consistently referred to as MG crises [13,16,40]. In our analysis of individuals with MG crises, the unpredictable and risk-laden nature of MG was emphasized. While some crises were associated with inciting events, primarily infections, more than three-quarters of MG crises were not associated with a specific trigger. Similarly, individuals with a history of MG crisis also commonly had a history of documented difficulties with MG treatments (43.8% of individuals with a history of MG crisis in this study). While the implications of some difficulties with treatment are relatively minor (e.g., stopping pyridostigmine due to excessive diarrhea or salivation), difficulties with receiving rescue therapies such as IVIg and plasmapheresis are potentially more serious. Neurologist documentation of medication difficulties in MG is consistent with published literature that discontinuations of MG medications due to adverse effects or poor response in the general population can run as high as 37% [7].

Our categorizations of neurologist documentation regarding risks and reluctance to treatment change, whether due to risk for deterioration or risk of treatment toxicity, identify potential drivers of clinical inertia in MG [31]. Clinical inertia has been described in the treatment of hypertension, diabetes, hyperlipidemia, and heart failure, and in a recent qualitative study of living with MG, performed by patient advocates from seven countries, treatment inertia emerged as a major theme [4,41-44]. As reflected in the selection of neurologist statements (Table 5) as well as the analysis of myasthenia crisis patients (Table 5), many MG medications are associated with substantial toxicity, and prescribing providers may be reluctant to start new agents that expose individuals with MG to unwanted side effects. Further research is needed to better understand provider and patient perspectives regarding MG-related clinical inertia, risks related to treatment, and opportunities to improve communication and care. 

Strengths

This study was the first application of NLP to examine the documentation of MG in neurologist clinical notes and provided an opportunity to examine physician language and the process of managing patients with MG, which is not feasible with coded datasets. This study sought to address the gap in knowledge of traditional observational research, which is routinely based on administrative (insurance claims) or structured clinical (EHR) data sources, and of qualitative studies of patient experience, which depend on patient-reported measures, by highlighting neurologist documentation of the clinical encounter. While MG is a rare condition, the clinical notes examined represented a wide spectrum of neurologists and patients from across the United States. The study results highlight the presence of important details contained within unstructured clinician notes (e.g., the presence of adverse effects to medications) as well as the variation in how providers describe their patients, symptoms, and outcomes, providing a baseline understanding of both the language of MG care as well as the limitations of existing documentation with regards to clinical subtyping and disease monitoring. Future research should seek to better understand physician needs and satisfaction with current documentation, leverage new digital capabilities to enhance documentation and overcome barriers to the use of patient-reported outcomes in routine MG care.

Limitations

The cross-sectional study design limited the ability to follow patient experience over time. Moreover, the dataset was limited to clinical notes only and thus provided little opportunity to assess the broader context of the clinical encounter. Future studies would benefit from using longitudinal data with additional data points to enable greater validation and learning. The NLP model did not seek to assess the timing of clinical events and symptoms in the clinical notes (i.e., prior versus current), limiting the interpretation of findings with regard to relationships between symptoms, treatment, and clinical events. The NLP development was heavily customized to this dataset to achieve high accuracy and precision and may not be generalizable to all physician notes. While this process supports the findings of this study, using the NLP rules for additional datasets would potentially return lower accuracy and precision. This study was limited to the notes that were available at the time of care and did not reflect the full set of information that was available to the practicing providers. As a result, clinical triggers for the MG crisis may have been present but not documented in the notes available in this study.

## Conclusions

Neurologist documentation highlights the challenges of managing MG, a heterogeneous disorder with unpredictable and sometimes life-threatening manifestations. While mortality related to MG has improved over time, neurologist documentation reflects the ongoing challenges of fluctuating illness. Novel therapies and care delivery improvements may help achieve optimal outcomes for MG by matching patients to therapies that control symptoms, prevent exacerbations and crises, and limit exposure to adverse medication effects. 

## Appendices

Supplemental materials A: Methods - NLP development

Text analysis consisted of 6 major steps including a) knowledge framework development, b) text preparation, c) tokenization and named entity recognition, d) assertion status detection, e) section-specific analysis, and f) model and textual complexity assessment.

The primary goal of the NLP model was to first to correctly identify the presence of concepts of interest within the progress notes and second to ensure correct interpretation of their meaning within the context of the sentence.  The analysis focused upon identifying textual representing the concept of interest as well as the surrounding cues within sentences indicating the status of a that concept (e.g., hypothetical, negation, family history, or assertion) (see Supplemental Table 1).  To further clarify the impact of cues upon the rules-based model performance, all sentences in all notes were characterized by the number of cues present in the sentence. 

Supplemental materials B - NLP assessment

Because the dataset contained large amounts of sentences with no mention of concepts of interest, the NLP model assertion status detection performance was assessed against randomly constructed datasets that included a mention of the concept of interest.  To ensure the NLP model was assessed across a broad spectrum of the relevant concepts, 16 datasets of 50 randomly selected sentence containing at least one mention of a concept of interest including dysphagia, ptosis, generalized myasthenia gravis, myasthenia gravis exacerbation or crisis, were generated.  For each of the concepts of interest, 4 datasets of 50 records were created that included 0-1 cue, 2 cues, 3 cues, and 4 or more cues.  Each of the datasets was then manually annotated using Prodigy, a proprietary software application, and the results compared with the NLP model applied to the same sentences. 

**NLP performance: **The rules-based NLP model accuracy was 0.97, precision 0.98, recall 0.98, and F1 score 0.98.  For sentences with 0-2 cues, model accuracy, precision, recall, and F1 score were all 1.0.  For sentences with 3 cues, model accuracy was 0.95, precision was 0.97, recall was 0.97, and F1 score was 0.97.  For sentences with 4 cues or more, model accuracy was 0.89, precision was 0.94, recall was 0.94, and F1 score was 0.92.

Table 6: Standard MG Therapies and MG Rescue therapies

**Table 6:** Standard MG Therapies and MG Rescue therapies *MG: Myasthenia Gravis, IVIg: Intravenous Immunoglobulin Therapy, PLEX: Plasma Exchange

Therapy Type	Therapy	Example Terms
Cholinesterase inhibitors	Pyridostigmine	“mestinon”, “pyridostigmine”
Corticosteroids	Corticosteroids	“prednisone”, “steroids”, “corticosteroid”, “medrol”
Other immunosuppressants	Azathioprine	“imuran”, “azathioprine”, “azasan”
	Mycophenolate mofetil	“cellcept”, “mycophenolate”
	Methotrexate	“reditrex”, “methotrexate”, “mtx”
	Tacrolimus	“tacrolimus”, “astagraf”, “prograf”
Monoclonal antibody	Rituximab	“rituxan”, “rituximab”
	Eculizumab	“soliris”, “eculizumab”
Neontal Fc receptor antagonist	Efgartigimod	“vyvgart”, “efgartigimod”
MG Rescue therapies	Plasmapheresis	“plasmapheresis”, “plasma exchange”, “PLEX”
	IVIg	“IVIG”, “privigen”

Table 7: Terms and Phrases normalized by Study Concepts

**Table 7:** Terms and Phrases normalized by Study Concepts

Type	Normalized_Concept	Text
CN	anti_musk_seropositive	anti-musk positive
CN	anti_musk_seropositive	anti-musk seropositive
CN	anti_musk_seropositive	musk-antibody positive
CN	anti_musk_seropositive	seropositive anti-musk
CN	antibody_positive	antibody positive
CN	antibody_positive	positive antibody
CN	bulbar_weakness	bulbar complaints
CN	bulbar_weakness	bulbar component
CN	bulbar_weakness	bulbar dysfunction
CN	bulbar_weakness	bulbar findings
CN	bulbar_weakness	bulbar in nature
CN	bulbar_weakness	bulbar issues
CN	bulbar_weakness	bulbar manifestations
CN	bulbar_weakness	bulbar muscle weakness
CN	bulbar_weakness	bulbar ocular symptoms
CN	bulbar_weakness	bulbar palsy
CN	bulbar_weakness	bulbar signs
CN	bulbar_weakness	bulbar symptom
CN	bulbar_weakness	bulbar symptoms
CN	bulbar_weakness	bulbar weakness
CN	bulbar_weakness	bulbar/ocular dysfunction
CN	bulbar_weakness	bulbar/ocular symptoms
CN	bulbar_weakness	fatigue of the bulbar muscles
CN	bulbar_weakness	ocular bulbar dysfunction
CN	bulbar_weakness	ocular bulbar signs
CN	bulbar_weakness	ocular bulbar symptoms
CN	bulbar_weakness	symptoms have been primarily bulbar
CN	bulbar_weakness	weak bulbar function
CN	bulbar_weakness	weakness of the bulbar
CN	choking	choke
CN	choking	choked
CN	choking	chokes
CN	choking	choking
CN	diaphragm_weakness	diaphragm weakness
CN	diaphragm_weakness	diaphragmatic weakness
CN	diaphragm_weakness	fatigability of the diaphragm
CN	diaphragm_weakness	respiratory muscle weakness
CN	diaphragm_weakness	weakness of the diaphragm
CN	diplopia	diploipia
CN	diplopia	diplopia
CN	diplopia	diploplia
CN	diplopia	double vision
CN	diplopia	paroxysmal horizontal diplopia
CN	dysarthria	dyarthria
CN	dysarthria	dysarthria
CN	dysarthria	dysarthric
CN	dysarthria	dysartris
CN	dysarthria	speech is weak
CN	dysarthria	voice is weak
CN	dysarthria	voice weak
CN	dysphagia	chewing is weak
CN	dysphagia	dysphagia
CN	dysphagia	weakness on chewing
CN	dysphasia	dysphasia
CN	dyspnea	breathlessness
CN	dyspnea	dyspna
CN	dyspnea	dyspnea
CN	dyspnea	dyspneic
CN	dyspnea	dyspnic
CN	dyspnea	short of air
CN	dyspnea	short of breath
CN	dyspnea	shortness breath
CN	dyspnea	shortness of air
CN	dyspnea	shortness of breath
CN	dyspnea	shortness of breathing
CN	dyspnea	sob
CN	dyspnea_at_rest	dyspnea at rest
CN	dyspnea_at_rest	dyspneic at rest
CN	dyspnea_at_rest	short of breath at rest
CN	dyspnea_at_rest	shortness of breath at rest
CN	dyspnea_on_exertion	doe
CN	dyspnea_on_exertion	dyspnea on exertion
CN	dyspnea_on_exertion	dyspnea with exertion
CN	dyspnea_on_exertion	dyspnea, worse on exertion
CN	dyspnea_on_exertion	dyspnea, worse with exertion
CN	dyspnea_on_exertion	exertional dyspnea
CN	dyspnea_on_exertion	exertional shortness of breath
CN	dyspnea_on_exertion	out of breath with exertion
CN	dyspnea_on_exertion	short of breath on exertion
CN	dyspnea_on_exertion	short of breath with exertion
CN	dyspnea_on_exertion	short of breath with minimal exertion
CN	dyspnea_on_exertion	shortness of breath on exertion
CN	dyspnea_on_exertion	shortness of breath with exertion
CN	dyspnea_on_exertion	shortness of breath with minimal exertion
CN	dyspnea_on_exertion	sob on exertion
CN	extremity_weakness	appendicular fatigue
CN	extremity_weakness	appendicular weakness
CN	extremity_weakness	arm and leg weakness
CN	extremity_weakness	arm or leg weakness
CN	extremity_weakness	extremities weakness
CN	extremity_weakness	extremity is weak
CN	extremity_weakness	extremity plegia
CN	extremity_weakness	extremity was weak
CN	extremity_weakness	extremity weakness
CN	extremity_weakness	limb muscle fatigue
CN	extremity_weakness	limb weakness
CN	extremity_weakness	peripheral weakness
CN	extremity_weakness	proximal extremity weakness
CN	extremity_weakness	proximal limb weakness
CN	extremity_weakness	upper and lower extremity weakness
CN	extremity_weakness	upper or lower extremity weakness
CN	extremity_weakness	weakness in an arm or leg
CN	extremity_weakness	weakness in arms or legs
CN	extremity_weakness	weakness in her arms and legs
CN	extremity_weakness	weakness in his arms and legs
CN	extremity_weakness	weakness in the arms and legs
CN	extremity_weakness	weakness in the arms and the legs
CN	extremity_weakness	weakness in the arms or legs
CN	extremity_weakness	weakness in the upper and lower extremities
CN	extremity_weakness	weakness in the upper and lower extremity
CN	extremity_weakness	weakness of arms or legs
CN	extremity_weakness	weakness of her arms or legs
CN	extremity_weakness	weakness of his arms or legs
CN	extremity_weakness	weakness of the arms or legs
CN	extremity_weakness	weakness of the limbs
CN	face_weakness	bifacial palsy
CN	face_weakness	bifacial weakness
CN	face_weakness	face weakness
CN	face_weakness	facial droop
CN	face_weakness	facial drooping
CN	face_weakness	facial motor weakness
CN	face_weakness	facial muscle is weak
CN	face_weakness	facial muscle weakness
CN	face_weakness	facial muscles are weak
CN	face_weakness	facial palsies
CN	face_weakness	facial palsy
CN	face_weakness	facial paresis
CN	face_weakness	facial weak
CN	face_weakness	facial weakness
CN	face_weakness	frontalis weakness
CN	face_weakness	mouth weakness
CN	face_weakness	weakness in face
CN	face_weakness	weakness in face muscles
CN	face_weakness	weakness in facial muscles
CN	face_weakness	weakness in her face
CN	face_weakness	weakness in his face
CN	face_weakness	weakness in the face
CN	face_weakness	weakness in the muscles of facial
CN	face_weakness	weakness of the face
CN	face_weakness	weakness of the facial
CN	fatigue	fatige
CN	fatigue	fatigue
CN	fatigue	fatigued
CN	fatigue	fatiguing
CN	fatigue	fatigure
CN	fatigue	lethargic
CN	fatigue	lethargy
CN	fatigue	malaise
CN	fatigue	tired
CN	fatigue	tiredness
CN	hoarseness	hoarse
CN	hoarseness	hoarse speech
CN	hoarseness	hoarse voice
CN	hoarseness	hoarseness
CN	hoarseness	hoarseness in her speech
CN	hoarseness	hoarseness in his speech
CN	hoarseness	hoarseness in his voice
CN	hoarseness	hoarseness is her voice
CN	hoarseness	hoarseness of her speech
CN	hoarseness	hoarseness of her voice
CN	hoarseness	hoarseness of his speech
CN	hoarseness	hoarseness of his voice
CN	hoarseness	hoarseness of the speech
CN	hoarseness	hoarseness of the voice
CN	hoarseness	hoarseness of voice
CN	hoarseness	hoarseness to her speech
CN	hoarseness	hoarseness to her voice
CN	hoarseness	hoarseness to his speech
CN	hoarseness	hoarseness to his voice
CN	hoarseness	hoarsness
CN	hoarseness	horseness
CN	hoarseness	lost her voice
CN	hoarseness	lost his voice
CN	hoarseness	speech hoarseness
CN	hoarseness	speech is hoarse
CN	hoarseness	speech is somewhat hoarse
CN	hoarseness	speech sounds hoarse
CN	hoarseness	voice and hoarseness
CN	hoarseness	voice became hoarse
CN	hoarseness	voice becomes hoarse
CN	hoarseness	voice gets hoarse
CN	hoarseness	voice has been hoarse
CN	hoarseness	voice hoarseness
CN	hoarseness	voice is hoarse
CN	hoarseness	voice, hoarseness
CN	jaw_weakness	jaw drop
CN	jaw_weakness	jaw fatigability
CN	jaw_weakness	jaw fatigue
CN	jaw_weakness	jaw fatiguing
CN	jaw_weakness	jaw feels weak
CN	jaw_weakness	jaw gets tired
CN	jaw_weakness	jaw muscle weakness
CN	jaw_weakness	jaw tiredness
CN	jaw_weakness	jaw weakness
CN	jaw_weakness	masseter weakness
CN	jaw_weakness	tired in her jaw
CN	jaw_weakness	tired in his jaw
CN	jaw_weakness	weakness in her jaw
CN	jaw_weakness	weakness in his jaw
CN	jaw_weakness	weakness of jaw
CN	jaw_weakness	weakness of the jaw
CN	lower_extremity_weakness	leg weakness
CN	lower_extremity_weakness	legs have been feeling weak
CN	lower_extremity_weakness	lower extremity weakness
CN	lower_extremity_weakness	lower limb weakness
CN	lower_extremity_weakness	weaknes in right lower extremity
CN	lower_extremity_weakness	weakness in either lower extremity
CN	lower_extremity_weakness	weakness in her legs
CN	lower_extremity_weakness	weakness in her lower extremities
CN	lower_extremity_weakness	weakness in her lower extremity
CN	lower_extremity_weakness	weakness in her lower limb
CN	lower_extremity_weakness	weakness in her lower limbs
CN	lower_extremity_weakness	weakness in his legs
CN	lower_extremity_weakness	weakness in his lower extremities
CN	lower_extremity_weakness	weakness in his lower extremity
CN	lower_extremity_weakness	weakness in his lower limb
CN	lower_extremity_weakness	weakness in his lower limbs
CN	lower_extremity_weakness	weakness in left lower extremity
CN	lower_extremity_weakness	weakness in the left leg
CN	lower_extremity_weakness	weakness in the left lower extremity
CN	lower_extremity_weakness	weakness in the legs
CN	lower_extremity_weakness	weakness in the lower extremities
CN	lower_extremity_weakness	weakness in the lower extremity
CN	lower_extremity_weakness	weakness in the lower limb
CN	lower_extremity_weakness	weakness in the lower limbs
CN	lower_extremity_weakness	weakness in the right leg
CN	lower_extremity_weakness	weakness in the right lower extremity
CN	lower_extremity_weakness	weakness in tibialis
CN	lower_extremity_weakness	weakness of the legs
CN	myasthenia_gravis	myasthenia
CN	myasthenia_gravis	myasthenia gravis
CN	myasthenia_gravis	myasthenic
CN	myasthenia_gravis_bulbar	bulbar and ocular myasthenia gravis
CN	myasthenia_gravis_bulbar	bulbar dysfunction myasthenia gravis
CN	myasthenia_gravis_bulbar	bulbar myasthenia
CN	myasthenia_gravis_bulbar	bulbar myasthenia gravis
CN	myasthenia_gravis_bulbar	bulbar ocular myasthenia gravis
CN	myasthenia_gravis_bulbar	bulbar predominance
CN	myasthenia_gravis_bulbar	bulbar predominant
CN	myasthenia_gravis_bulbar	bulbar predominant myasthenia gravis
CN	myasthenia_gravis_bulbar	bulbar/ocular myasthenia
CN	myasthenia_gravis_bulbar	bulbar/ocular myasthenia gravis
CN	myasthenia_gravis_bulbar	myasthenia gravis with a bulbar
CN	myasthenia_gravis_bulbar	myasthenia gravis with bulbar
CN	myasthenia_gravis_bulbar	myasthenia gravis, bulbar
CN	myasthenia_gravis_bulbar	myasthenia gravis, ocular bulbar
CN	myasthenia_gravis_bulbar	myasthenia, bulbar
CN	myasthenia_gravis_bulbar	ocular bulbar myasthenia gravis
CN	myasthenia_gravis_bulbar	ocular/bulbar myasthenia gravis
CN	myasthenia_gravis_bulbar	ocular/bulbar predominance
CN	myasthenia_gravis_bulbar	ocular/bulbar predominant
CN	myasthenia_gravis_bulbar	oculopharyngeal myasthenia gravis
CN	myasthenia_gravis_bulbar	ophthalmic/bulbar myasthenia
CN	myasthenia_gravis_bulbar	ophthalmic/bulbar myasthenia gravis
CN	myasthenia_gravis_bulbar	pharyngeal myasthenia gravis
CN	myasthenia_gravis_bulbar_seronegative	antibody negative bulbar myasthenia gravis
CN	myasthenia_gravis_bulbar_seronegative	bulbar seronegative myasthenia gravis
CN	myasthenia_gravis_bulbar_seronegative	seronegative bulbar myasthenia gravis
CN	myasthenia_gravis_bulbar_seropositive	antibody positive bulbar myasthenia
CN	myasthenia_gravis_bulbar_seropositive	antibody positive bulbar myasthenia gravis
CN	myasthenia_gravis_bulbar_seropositive	bulbar seropositive myasthenia gravis
CN	myasthenia_gravis_bulbar_seropositive	seropositive bulbar myasthenia gravis
CN	myasthenia_gravis_crisis	crisis of myasthenia gravis
CN	myasthenia_gravis_crisis	myasthenia crisis
CN	myasthenia_gravis_crisis	myasthenia gravis crisis
CN	myasthenia_gravis_crisis	myasthenia gravis in crisis
CN	myasthenia_gravis_crisis	myasthenia gravis, acute crisis
CN	myasthenia_gravis_crisis	myasthenia gravis/crisis
CN	myasthenia_gravis_crisis	myasthenic crisis
CN	myasthenia_gravis_exacerbation	exacerbation from myasthenia gravis
CN	myasthenia_gravis_exacerbation	exacerbation of her myasthenia
CN	myasthenia_gravis_exacerbation	exacerbation of her myasthenia gravis
CN	myasthenia_gravis_exacerbation	exacerbation of his myasthenia
CN	myasthenia_gravis_exacerbation	exacerbation of his myasthenia gravis
CN	myasthenia_gravis_exacerbation	exacerbation of myasthenia
CN	myasthenia_gravis_exacerbation	exacerbation of myasthenia gravis
CN	myasthenia_gravis_exacerbation	exacerbation of the myasthenia
CN	myasthenia_gravis_exacerbation	exacerbation of the myasthenia gravis
CN	myasthenia_gravis_exacerbation	flare of myasthenia gravis
CN	myasthenia_gravis_exacerbation	mg exacerbation
CN	myasthenia_gravis_exacerbation	myasthenia exacerbation
CN	myasthenia_gravis_exacerbation	myasthenia flare
CN	myasthenia_gravis_exacerbation	myasthenia gravis acute exacerbation
CN	myasthenia_gravis_exacerbation	myasthenia gravis and exacerbation
CN	myasthenia_gravis_exacerbation	myasthenia gravis attack
CN	myasthenia_gravis_exacerbation	myasthenia gravis exacerbation
CN	myasthenia_gravis_exacerbation	myasthenia gravis exacerbations
CN	myasthenia_gravis_exacerbation	myasthenia gravis flare
CN	myasthenia_gravis_exacerbation	myasthenia gravis flare up
CN	myasthenia_gravis_exacerbation	myasthenia gravis flareup
CN	myasthenia_gravis_exacerbation	myasthenia gravis in exacerbation
CN	myasthenia_gravis_exacerbation	myasthenia gravis relapse
CN	myasthenia_gravis_exacerbation	myasthenia gravis with acute exacerbation
CN	myasthenia_gravis_exacerbation	myasthenia gravis with exacerbation
CN	myasthenia_gravis_exacerbation	myasthenia gravis with recent exacerbation
CN	myasthenia_gravis_exacerbation	myasthenia gravis, acute exacerbation
CN	myasthenia_gravis_exacerbation	myasthenia gravis, in exacerbation
CN	myasthenia_gravis_exacerbation	myasthenia gravis, with exacerbation
CN	myasthenia_gravis_exacerbation	myasthenic exacerbation
CN	myasthenia_gravis_exacerbation	myasthenic exacerbations
CN	myasthenia_gravis_exacerbation	myasthenic flare
CN	myasthenia_gravis_exacerbation	myasthenic flareup
CN	myasthenia_gravis_exacerbation	myasthenic gravis exacerbation
CN	myasthenia_gravis_exacerbation	myasthenic relapse
CN	myasthenia_gravis_generalized	generalized and ocular myasthenia gravis
CN	myasthenia_gravis_generalized	generalized form of myasthenia gravis
CN	myasthenia_gravis_generalized	generalized myasthenia
CN	myasthenia_gravis_generalized	generalized myasthenia gravis
CN	myasthenia_gravis_generalized	generalized myasthenic disorder
CN	myasthenia_gravis_generalized	generalized ocular myasthenia gravis
CN	myasthenia_gravis_generalized	myasthenia gravis generalized
CN	myasthenia_gravis_generalized	myasthenia gravis, generalized
CN	myasthenia_gravis_generalized	myasthenia gravis, systemic
CN	myasthenia_gravis_generalized	myasthenia is generalized
CN	myasthenia_gravis_generalized	myasthenia, generalized
CN	myasthenia_gravis_generalized	systemic myasthenia gravis
CN	myasthenia_gravis_generalized_seronegative	antibody negative generalized myasthenia gravis
CN	myasthenia_gravis_generalized_seronegative	generalized myasthenia gravis seronegative
CN	myasthenia_gravis_generalized_seronegative	generalized myasthenia gravis, seronegative
CN	myasthenia_gravis_generalized_seronegative	generalized seronegative myasthenia gravis
CN	myasthenia_gravis_generalized_seronegative	myasthenia gravis, generalized seronegative
CN	myasthenia_gravis_generalized_seronegative	seronegative generalized myasthenia gravis
CN	myasthenia_gravis_generalized_seronegative	seronegative myasthenia gravis, generalized
CN	myasthenia_gravis_generalized_seropositive	antibody positive generalized myasthenia gravis
CN	myasthenia_gravis_generalized_seropositive	generalized myasthenia gravis seropositive
CN	myasthenia_gravis_generalized_seropositive	generalized myasthenia gravis, seropositive
CN	myasthenia_gravis_generalized_seropositive	generalized seropositive myasthenia gravis
CN	myasthenia_gravis_generalized_seropositive	myasthenia gravis, generalized seropositive
CN	myasthenia_gravis_generalized_seropositive	seropositive autoimmune myasthenia gravis
CN	myasthenia_gravis_generalized_seropositive	seropositive generalized autoimmune myasthenia gravis
CN	myasthenia_gravis_generalized_seropositive	seropositive generalized myasthenia gravis
CN	myasthenia_gravis_musk	anti-musk antibody myasthenia
CN	myasthenia_gravis_musk	musk antibody positive myasthenia gravis
CN	myasthenia_gravis_musk	musk myasthenia
CN	myasthenia_gravis_musk	musk positive myasthenia gravis
CN	myasthenia_gravis_musk	musk-positive antibody myasthenia
CN	myasthenia_gravis_musk	musk-positive myasthenia gravis
CN	myasthenia_gravis_musk	myasthenia gravis associated with antibodies to musk
CN	myasthenia_gravis_musk	myasthenia gravis with antibodies to musk
CN	myasthenia_gravis_ocular	myasthenia, ocular
CN	myasthenia_gravis_ocular	ocular myasthenia
CN	myasthenia_gravis_ocular	ocular myasthenia gravis
CN	myasthenia_gravis_ocular	ocular predominance
CN	myasthenia_gravis_ocular	ocular predominant
CN	myasthenia_gravis_ocular	ocular predominant myasthenia
CN	myasthenia_gravis_ocular	ocular predominant myasthenia gravis
CN	myasthenia_gravis_ocular	ophthalmic myasthenia gravis
CN	myasthenia_gravis_ocular_seronegative	antibody negative ocular myasthenia gravis
CN	myasthenia_gravis_ocular_seronegative	antibody-negative ocular myasthenia
CN	myasthenia_gravis_ocular_seronegative	antibody-negative ocular myasthenia gravis
CN	myasthenia_gravis_ocular_seronegative	ocular antibody negative myasthenia gravis
CN	myasthenia_gravis_ocular_seronegative	ocular myasthenia seronegative
CN	myasthenia_gravis_ocular_seronegative	ocular seronegative myasthenia gravis
CN	myasthenia_gravis_ocular_seronegative	seronegative ocular myasthenia gravis
CN	myasthenia_gravis_ocular_seronegative	seropositive myasthenia gravis, generalized
CN	myasthenia_gravis_ocular_seropositive	antibody positive ocular myasthenia gravis
CN	myasthenia_gravis_ocular_seropositive	antibody-positive ocular myasthenia
CN	myasthenia_gravis_ocular_seropositive	antibody-positive ocular myasthenia gravis
CN	myasthenia_gravis_ocular_seropositive	ocular antibody positive myasthenia gravis
CN	myasthenia_gravis_ocular_seropositive	ocular myasthenia gravis antibody positive
CN	myasthenia_gravis_ocular_seropositive	ocular myasthenia seropositive
CN	myasthenia_gravis_ocular_seropositive	ocular seropositive myasthenia gravis
CN	myasthenia_gravis_ocular_seropositive	seropositive ocular myasthenia gravis
CN	myasthenia_gravis_overlap_syndrome	myasthenia gravis overlap syndrome
CN	myasthenia_gravis_seronegative	acetylcholine receptor negative myasthenia gravis
CN	myasthenia_gravis_seronegative	antibody-negative myasthenia gravis
CN	myasthenia_gravis_seronegative	autoimmune myasthenia gravis, seronegative
CN	myasthenia_gravis_seronegative	myasthenia gravis antibody negative
CN	myasthenia_gravis_seronegative	myasthenia gravis seronegative
CN	myasthenia_gravis_seronegative	myasthenia gravis, antibody negative
CN	myasthenia_gravis_seronegative	myasthenia gravis, seronegative
CN	myasthenia_gravis_seronegative	myasthenia gravis/seronegative
CN	myasthenia_gravis_seronegative	receptor negative generalized myasthenia gravis
CN	myasthenia_gravis_seronegative	receptor negative myasthenia gravis
CN	myasthenia_gravis_seronegative	seronegative myasthenia
CN	myasthenia_gravis_seronegative	seronegative myasthenia gravis
CN	myasthenia_gravis_seropositive	acetylcholine receptor antibody myasthenia
CN	myasthenia_gravis_seropositive	acetylcholine receptor antibody positive
CN	myasthenia_gravis_seropositive	acetylcholine receptor antibody positive myasthenia gravis
CN	myasthenia_gravis_seropositive	acetylcholine receptor antibody positivity
CN	myasthenia_gravis_seropositive	acetylcholine receptor positive myasthenia
CN	myasthenia_gravis_seropositive	acetylcholine receptor positive myasthenia gravis
CN	myasthenia_gravis_seropositive	acetylcholine-receptor antibody positive myasthenia gravis
CN	myasthenia_gravis_seropositive	antibody positive myasthenia gravis
CN	myasthenia_gravis_seropositive	antibody-positive myasthenia gravis
CN	myasthenia_gravis_seropositive	autoimmune myasthenia gravis, seropositive
CN	myasthenia_gravis_seropositive	myasthenia gravis antibody positive
CN	myasthenia_gravis_seropositive	myasthenia gravis autoimmune, seropositive
CN	myasthenia_gravis_seropositive	myasthenia gravis seropositive
CN	myasthenia_gravis_seropositive	myasthenia gravis with positive antibodies
CN	myasthenia_gravis_seropositive	myasthenia gravis, antibody positive
CN	myasthenia_gravis_seropositive	myasthenia gravis, seropositive
CN	myasthenia_gravis_seropositive	myasthenia gravis/seropositive
CN	myasthenia_gravis_seropositive	positive antibody testing for myasthenia
CN	myasthenia_gravis_seropositive	receptor positive generalized myasthenia gravis
CN	myasthenia_gravis_seropositive	receptor positive myasthenia gravis
CN	myasthenia_gravis_seropositive	seropositive for myasthenia gravis
CN	myasthenia_gravis_seropositive	seropositive myasthenia
CN	myasthenia_gravis_seropositive	seropositive myasthenia gravis
CN	neck_weakness	head drop
CN	neck_weakness	neck drop
CN	neck_weakness	neck extension weakness
CN	neck_weakness	neck extensor weakness
CN	neck_weakness	neck flexion weakness
CN	neck_weakness	neck flexor weakness
CN	neck_weakness	neck is weak
CN	neck_weakness	neck muscle weakness
CN	neck_weakness	neck weakness
CN	neck_weakness	weak neck
CN	neck_weakness	weakness in her neck
CN	neck_weakness	weakness in his neck
CN	neck_weakness	weakness in neck
CN	neck_weakness	weakness in neck extension
CN	neck_weakness	weakness in neck flexion
CN	neck_weakness	weakness in the neck
CN	neck_weakness	weakness of her neck flexors
CN	neck_weakness	weakness of his neck flexors
CN	neck_weakness	weakness of neck
CN	neck_weakness	weakness of the neck
CN	neck_weakness	weakness of the neck extensors
CN	neck_weakness	weakness of the neck flexor
CN	neck_weakness	weakness of the neck flexors
CN	neck_weakness	weakness on neck extension
CN	neck_weakness	weakness on neck flexion
CN	oropharyngeal_weakness	oropharyngeal weakness
CN	oropharyngeal_weakness	pharynx is weak
CN	oropharyngeal_weakness	throat weakness
CN	ptosis	droopiness of her eyelid
CN	ptosis	droopiness of her eyelids
CN	ptosis	droopiness of her left eye
CN	ptosis	droopiness of her left eyelid
CN	ptosis	droopiness of her right eye
CN	ptosis	droopiness of her right eyelid
CN	ptosis	droopiness of his eyelid
CN	ptosis	droopiness of his eyelids
CN	ptosis	droopiness of his left eye
CN	ptosis	droopiness of his left eyelid
CN	ptosis	droopiness of his right eye
CN	ptosis	droopiness of his right eyelid
CN	ptosis	droopiness of the eyelid
CN	ptosis	droopiness of the eyelids
CN	ptosis	drooping eyelid
CN	ptosis	drooping eyelids
CN	ptosis	drooping in eyelid
CN	ptosis	drooping in eyelids
CN	ptosis	drooping of both eyelids
CN	ptosis	drooping of her eyelid
CN	ptosis	drooping of her eyelids
CN	ptosis	drooping of her eyes
CN	ptosis	drooping of his eyelid
CN	ptosis	drooping of his eyelids
CN	ptosis	drooping of his eyes
CN	ptosis	drooping of the eyelid
CN	ptosis	drooping of the eyelids
CN	ptosis	drooping of the eyes
CN	ptosis	drooping of the left eye
CN	ptosis	drooping of the left eyelid
CN	ptosis	drooping of the right eye
CN	ptosis	drooping of the right eyelid
CN	ptosis	droopy eye
CN	ptosis	droopy eyelid
CN	ptosis	droopy eyelids
CN	ptosis	droopy eyes
CN	ptosis	droopy left eyelid
CN	ptosis	droopy lid
CN	ptosis	droopy lids
CN	ptosis	droopy right eyelid
CN	ptosis	eye droopiness
CN	ptosis	eyelid droop
CN	ptosis	eyelid drooping
CN	ptosis	eyelid was droopy
CN	ptosis	eyelids droop
CN	ptosis	eyelids drooping
CN	ptosis	eyelids weakness
CN	ptosis	eyes are droopy
CN	ptosis	eyes droop
CN	ptosis	ptosis
CN	respiratory_disease	respiratory disease
CN	respiratory_disease	respiratory illness
CN	respiratory_distress	respiratory compromise
CN	respiratory_distress	respiratory distress
CN	respiratory_distress	respiratory insufficiency
CN	respiratory_distress	respiratory weakness
CN	respiratory_distress	unable to breath
CN	respiratory_failure	hypoxic rf
CN	respiratory_failure	respiratory arrest
CN	respiratory_failure	respiratory failure
CN	respiratory_failure	respiratory faluire
CN	tongue_weakness	lingual muscle weakness
CN	tongue_weakness	tongue weakness
CN	tongue_weakness	weakness of her tongue
CN	tongue_weakness	weakness of his tongue
CN	tongue_weakness	weakness of the tongue
CN	upper_extremity_weakness	arm weakness
CN	upper_extremity_weakness	arms have been feeling weak
CN	upper_extremity_weakness	hand weakness
CN	upper_extremity_weakness	left arm weakness
CN	upper_extremity_weakness	left triceps weakness
CN	upper_extremity_weakness	right arm weakness
CN	upper_extremity_weakness	right triceps weakness
CN	upper_extremity_weakness	upper extremity weakness
CN	upper_extremity_weakness	weak grip
CN	upper_extremity_weakness	weakness in her arms
CN	upper_extremity_weakness	weakness in her hands
CN	upper_extremity_weakness	weakness in her upper extremities
CN	upper_extremity_weakness	weakness in her upper extremity
CN	upper_extremity_weakness	weakness in her upper limb
CN	upper_extremity_weakness	weakness in her upper limbs
CN	upper_extremity_weakness	weakness in his arms
CN	upper_extremity_weakness	weakness in his hands
CN	upper_extremity_weakness	weakness in his upper extremities
CN	upper_extremity_weakness	weakness in his upper extremity
CN	upper_extremity_weakness	weakness in his upper limb
CN	upper_extremity_weakness	weakness in his upper limbs
CN	upper_extremity_weakness	weakness in the arms
CN	upper_extremity_weakness	weakness in the hands
CN	upper_extremity_weakness	weakness in the left upper extremity
CN	upper_extremity_weakness	weakness in the right upper extremity
CN	upper_extremity_weakness	weakness in the upper extremities
CN	upper_extremity_weakness	weakness in the upper extremity
CN	upper_extremity_weakness	weakness in the upper limb
CN	upper_extremity_weakness	weakness in the upper limbs
CN	upper_extremity_weakness	weakness of her arms
CN	upper_extremity_weakness	weakness of his arms
CN	upper_extremity_weakness	weakness of the arms
CN	weakness	fatigable weakness
CN	weakness	plegia
CN	weakness	weak
CN	weakness	weakened
CN	weakness	weakenss
CN	weakness	weaker
CN	weakness	weakess
CN	weakness	weaknees
CN	weakness	weaknes
CN	weakness	weakness
CN	weakness	weaknesss
CN	weakness	wekaness
CN	weakness_focal	focal paresis
CN	weakness_focal	focal weakness
CN	weakness_generalized	diffuse muscle weakness
CN	weakness_generalized	diffuse weakness
CN	weakness_generalized	feels weak all over
CN	weakness_generalized	general weakness
CN	weakness_generalized	generalized fatigability
CN	weakness_generalized	generalized fatigable weakness
CN	weakness_generalized	generalized muscle fatigability
CN	weakness_generalized	generalized muscle weakness
CN	weakness_generalized	generalized muscular weakness
CN	weakness_generalized	generalized sense of weakness
CN	weakness_generalized	generalized weakness
CN	weakness_generalized	generally weak
CN	weakness_generalized	global weakness
CN	weakness_generalized	overall weakness
CN	weakness_generalized	systemic weakness
CN	weakness_generalized	weakness is generalized
CN	weakness_generalized	whole body was weak
CN	weakness_left	left side of body weakness
CN	weakness_left	left-sided weakness
CN	weakness_muscle	motor weakness
CN	weakness_muscle	muscle fatigue
CN	weakness_muscle	muscle weakness
CN	weakness_muscle	muscular weakness
CN	weakness_muscle	proximal weakness
CN	weakness_muscle	weakness in her muscles
CN	weakness_muscle	weakness in his muscles
CN	weakness_muscle	weakness of muscles
CN	weakness_right	right side of body weakness
CN	weakness_right	right-sided weakness
CRISIS	crisiscue	crises
CRISIS	crisiscue	crisis
EXACERBATIONCUE	exacerbationcue	decompensated
EXACERBATIONCUE	exacerbationcue	decompensation
EXACERBATIONCUE	exacerbationcue	deteriorated
EXACERBATIONCUE	exacerbationcue	deterioration
EXACERBATIONCUE	exacerbationcue	exacerbate
EXACERBATIONCUE	exacerbationcue	exacerbated
EXACERBATIONCUE	exacerbationcue	exacerbation
EXACERBATIONCUE	exacerbationcue	exacerbations
EXACERBATIONCUE	exacerbationcue	flare
EXACERBATIONCUE	exacerbationcue	flares
EXACERBATIONCUE	exacerbationcue	flareup
EXACERBATIONCUE	exacerbationcue	out of control
EXACERBATIONCUE	exacerbationcue	poorly controlled
EXACERBATIONCUE	exacerbationcue	relapse
EXACERBATIONCUE	exacerbationcue	relapsed
EXACERBATIONCUE	exacerbationcue	relapses
EXACERBATIONCUE	exacerbationcue	relapsing
EXACERBATIONCUE	exacerbationcue	uncontrolled
EXACERBATIONCUE	exacerbationcue	unstable
LAB	acetylcholine_receptor_antibody	acetylcholine antibodies
LAB	acetylcholine_receptor_antibody	acetylcholine antibody
LAB	acetylcholine_receptor_antibody	acetylcholine antibody panel
LAB	acetylcholine_receptor_antibody	acetylcholine binding antibodies
LAB	acetylcholine_receptor_antibody	acetylcholine binding antibody
LAB	acetylcholine_receptor_antibody	acetylcholine binding, blocking, and modulating antibodies
LAB	acetylcholine_receptor_antibody	acetylcholine binding, blocking, and modulating antibody
LAB	acetylcholine_receptor_antibody	acetylcholine blocking antibody
LAB	acetylcholine_receptor_antibody	acetylcholine modulating antibodies
LAB	acetylcholine_receptor_antibody	acetylcholine modulating antibody
LAB	acetylcholine_receptor_antibody	acetylcholine receptor
LAB	acetylcholine_receptor_antibody	acetylcholine receptor antibodies
LAB	acetylcholine_receptor_antibody	acetylcholine receptor antibody
LAB	acetylcholine_receptor_antibody	acetylcholine receptor binding antibodies
LAB	acetylcholine_receptor_antibody	acetylcholine receptor binding antibody
LAB	acetylcholine_receptor_antibody	acetylcholine receptor blocking antibodies
LAB	acetylcholine_receptor_antibody	acetylcholine receptor blocking antibody
LAB	acetylcholine_receptor_antibody	acetylcholine receptor modulating antibodies
LAB	acetylcholine_receptor_antibody	acetylcholine receptor modulating antibody
LAB	acetylcholine_receptor_antibody	acetylcholine receptor-binding antibody
LAB	acetylcholine_receptor_antibody	acetylcholine serum antibodies
LAB	acetylcholine_receptor_antibody	acetylcholine-receptor antibodies
LAB	acetylcholine_receptor_antibody	acetylcholine-receptor antibody
LAB	acetylcholine_receptor_antibody	acetylcholine-receptor antibody titers
LAB	acetylcholine_receptor_antibody	ach receptor
LAB	acetylcholine_receptor_antibody	ach receptor antibodies
LAB	acetylcholine_receptor_antibody	ach receptor antibody
LAB	acetylcholine_receptor_antibody	achr antibodies
LAB	acetylcholine_receptor_antibody	achr antibody
LAB	acetylcholine_receptor_antibody	anti-acetylcholine receptor antibody
LAB	acetylcholine_receptor_antibody	antiacetylcholine receptor antibody testing
LAB	acetylcholine_receptor_antibody	antibodies to acetylcholine receptor
LAB	acetylcholine_receptor_antibody	antibody to acetylcholine receptor
LAB	acetylcholine_receptor_antibody	binding, blocking and modulating antibodies
LAB	acetylcholine_receptor_antibody	binding, blocking, and modulating antibodies
LAB	mg_adl	mg adl
LAB	mg_adl	mg-adl
LAB	mg_adl	myasthenia gravis adl
LAB	mg_adl	myasthenia gravis adl score
LAB	musk_antibody	anti musk
LAB	musk_antibody	anti-musk
LAB	musk_antibody	antimusk antibodies
LAB	musk_antibody	anti-musk antibodies
LAB	musk_antibody	antimusk antibody
LAB	musk_antibody	anti-musk antibody
LAB	musk_antibody	musk
LAB	musk_antibody	musk antibodies
LAB	musk_antibody	musk antibody
LAB	musk_antibody	musk antibody panel
LAB	musk_antibody	musk test
WORSECUE	worseningcue	, worse early
WORSECUE	worseningcue	, worse in
WORSECUE	worseningcue	, worse on
WORSECUE	worseningcue	, worsening over
WORSECUE	worseningcue	, worsening toward
WORSECUE	worseningcue	, worsens with
WORSECUE	worseningcue	aggravate
WORSECUE	worseningcue	aggravated
WORSECUE	worseningcue	aggravates
WORSECUE	worseningcue	aggravating
WORSECUE	worseningcue	aggravation
WORSECUE	worseningcue	disease progression
WORSECUE	worseningcue	grown
WORSECUE	worseningcue	has gotten worse
WORSECUE	worseningcue	increasingly
WORSECUE	worseningcue	invade
WORSECUE	worseningcue	invaded
WORSECUE	worseningcue	invades
WORSECUE	worseningcue	invading
WORSECUE	worseningcue	invasion
WORSECUE	worseningcue	not doing well
WORSECUE	worseningcue	progressed
WORSECUE	worseningcue	progresses
WORSECUE	worseningcue	progressing
WORSECUE	worseningcue	progression
WORSECUE	worseningcue	progression of
WORSECUE	worseningcue	progression to
WORSECUE	worseningcue	progressive
WORSECUE	worseningcue	progressively
WORSECUE	worseningcue	rebound
WORSECUE	worseningcue	relapse of
WORSECUE	worseningcue	spread
WORSECUE	worseningcue	worse
WORSECUE	worseningcue	worse than
WORSECUE	worseningcue	worseing
WORSECUE	worseningcue	worsened
WORSECUE	worseningcue	worsenign
WORSECUE	worseningcue	worsenin
WORSECUE	worseningcue	worsening
WORSECUE	worseningcue	worsening of
WORSECUE	worseningcue	worsens
WORSECUE	worseningcue	worsethan
WORSECUE	worseningcue	worst
WORSECUE	worseningcue	, more
WORSECUE	worseningcue	, progressive
WORSECUE	worseningcue	, worse
WORSECUE	worseningcue	, worsening
WORSECUE	worseningcue	inceasing
WORSECUE	worseningcue	incr
WORSECUE	worseningcue	increase
WORSECUE	worseningcue	increased
WORSECUE	worseningcue	increaseing
WORSECUE	worseningcue	increasing
WORSECUE	worseningcue	more

Table 8: Examples of cue terms-phrases

**Table 8:** Examples of cue terms-phrases

Cue Type	Example Terms and Phrases
Break	“and that”, “complicated by”, “in regards to”
Hypothetical	“rule out”, “to avoid”, “if”, “differential diagnosis”
Negation	“denied”, “never”, “no history”
Family history	“family history”, “brother”, “grandmother”, “sibling”
Difficulty	“disturbance”, “impairment”, “inability”
Worsening	“worsening”, ”increasing”, “progression of”
Improving	“is getting better”, “responding well to”, “complete resolution of”
Normal	“undetectable”, “unremarkable”, “normal”
Abnormal	“elevated”, “positive”
Allergy-Adverse drug effect	“allergic”, “intolerant”

Table 9: Accuracy, Precision, Recall, and F1 scores for concepts by number of cues

**Table 9:** Accuracy, Precision, Recall, and F1 scores for concepts by number of cues *F1 score is the weighted average of recall and precision which gives a more useful understanding of a model’s performance especially with unbalanced datasets.  A perfect F1 score is 1 and a failed model would score 0.

Number of cues	Accuracy*	Precision*	Recall*	F1 score*
Any	0.96	0.98	0.98	0.97
0 to 1	1.00	1.00	1.00	1.00
2	1.00	1.00	1.00	1.00
3	0.952	0.971	0.971	0.971
4+	0.886	0.938	0.938	0.917

Table 10: Types of challenges or difficulties with specific MG therapies

**Table 10:** Types of challenges or difficulties with specific MG therapies *MG: Myasthenia Gravis, IVIg: Intravenous Immunoglobulin Therapy

Challenges or difficulty receiving specific MG therapy‡	
Pyridostigmine (adverse effects 20, limited response 5, interaction with comorbid conditions 3)	27 (19.7)
IVIg (adverse effects 8, limited response 9, interaction with comorbid conditions 4, poor availability at treatment facility 1, problems with insurance or prior authorization 2)	23 (16.7)
Corticosteroids (adverse effects 12, limited response 4, interaction with comorbid conditions 3)	19 (13.8)
Plasmapheresis (adverse effects 6, limited response 3, interaction with comorbid conditions 1, poor availability at treatment facility 2)	12 (8.8)
Azathioprine (adverse effects 9, limited response 2, interaction with comorbid condition 1, problems with insurance or prior authorization 1)	12 (8.8)
Mycophenolate (adverse effects 1)	1 (0.7)
Eculizumab (adverse effects 1)	1 (0.7)
Rituximab (adverse effects 1)	1 (0.7)

Figure 1: Definitions of accuracy, recall, precision, and F1 score using a confusion matrix
